# MP0250, a VEGF and HGF neutralizing DARPin^®^ molecule shows high anti-tumor efficacy in mouse xenograft and patient-derived tumor models

**DOI:** 10.18632/oncotarget.21738

**Published:** 2017-10-11

**Authors:** Ulrike Fiedler, Savira Ekawardhani, Andreas Cornelius, Pat Gilboy, Talitha R. Bakker, Ignacio Dolado, Michael T. Stumpp, Keith M. Dawson

**Affiliations:** ^1^ Molecular Partners AG, Schlieren, Switzerland; ^2^ Horizon Pharma, Dublin, Ireland; ^3^ Amgen (Europe) GmbH, Zug, Switzerland; ^4^ Roche, Basel, Switzerland

**Keywords:** VEGF, HGF, serum albumin, DARPin^®^, patient-derived xenograft

## Abstract

**Background:**

The VEGF/VEGFR and the HGF/cMET pathways are key mediators of the interplay of tumor cells and their microenvironment. However, inhibition of VEGF has been shown to produce only limited clinical benefit and inhibition of the activation of cMET by HGF has not translated into clinical benefit in pivotal trials. MP0250, a DARPin^®^ molecule that specifically inhibits both VEGF and HGF has been developed to explore the clinical potential of dual inhibition of these pathways.

**Results:**

MP0250 binding to VEGF and HGF inhibited downstream signalling through VEGFR2 and cMET resulting in inhibition of proliferation of VEGF- and HGF-dependent cells. Antitumor activity was demonstrated in VEGF- and HGF-dependent xenograft and syngeneic models with activity superior to that of individual VEGF- and HGF-blocking DARPin^®^ molecules. Combination therapy studies showed potentiation of the antitumor activity of chemotherapy and immunotherapy agents, including an anti-PD1 antibody.

**Materials and Methods:**

Potency of MP0250 was assessed in cellular models and in a variety of xenograft models as monotherapy or in combination with standard-of-care drugs.

**Conclusions:**

Dual inhibition of VEGF and HGF by MP0250 produced powerful single agent and combination antitumor activity. This, together with increasing understanding of the role of the HGF/cMET pathway in resistance to VEGF (and other agents), supports testing of MP0250 in the clinic.

## INTRODUCTION

The interplay of tumor cells and their microenvironment is crucial in the growth of solid tumors [1] and the VEGF/VEGFR and the HGF/cMET pathways are key mediators of such interaction [2, 3]. Nonclinical studies have shown that cMET can be upregulated to overcome VEGF resistance and that simultaneous inhibition of these pathways can overcome treatment resistance [4, 5]. More recently it has been shown that the pathways cross-regulate each other with cMET inhibition leading to upregulation of the VEGF pathway and inhibition of the VEGF pathway overcoming cMET-resistance in tumor models [6].

Individually, inhibitors of the VEGF and HGF/cMET pathways have had mixed success in the clinic. Inhibitors of the VEGF pathway (e.g. bevacizumab, sorafenib) are approved for anti-angiogenic treatment of various solid tumors [5]. However improvements in progression-free survival outcomes have not consistently translated into improved overall survival and all patients eventually develop resistance and progress [7, 8]. Resistance to bevacizumab therapy has been reported in preclinical models to be associated with amplification of cMET signaling [4, 9]. This highlights the need for improved anti-angiogenic therapy that can augment VEGF inhibition as well as inhibit the development of HGF-mediated resistance [4, 10].

Aberrant cMET/HGF expression is observed in numerous types of cancer and is associated with a poor prognosis [11]. It has been demonstrated in preclinical models of human cancer that aberrant cMET signaling occurs at significantly higher frequency following anti-VEGF [4] [12] [13]. In addition, cMET activation is a cause of resistance to other therapies, such as epidermal growth factor receptor (EGFR) inhibitor therapy [12].

However, despite the evidence indicating the importance of the cMET pathway in tumor growth and resistance, clinical trials of monoclonal antibodies targeting the cMET pathway have so far failed to show significant activity. For example, the HGF-targeted monoclonal antibody rilotumumab failed to show benefit when tested in combination with chemotherapy in a phase III clinical trial in patients with gastric or gastroesophageal junction cancer [14] and onartuzumab, a cMET targeted monovalent antibody, failed to show benefit when tested in combination with the EGFR inhibitor erlotinib in a phase III non-small-cell lung cancer trial [15]. The recently reported finding that rilotumumab is only a partial antagonist of HGF activation of cMET on conventional and primary patient-derived human gliomasphere lines could offer a plausible explanation for the failure of rilotumumab [16]. However, for onartuzumab, the reason for failure in the pivotal trial seems more likely to be inappropriate patient selection [15, 17]. On the other hand, as it has been reported that survival in the pivotal trials was worse in rilotumumab- and onatuzumab-treated patients than in the controls, a potential explanation could be that inhibition of cMET activation leads to upregulation of an escape mechanism, for instance the VEGF pathway [6].

Only very limited clinical investigation of combined treatment with VEGF and HGF/cMET inhibitors has been reported: a phase Ib trial showed the safety of combining bevacizumab with rilotumumab in cancer patients [18] and a phase II trial of bevacizumab and onartuzumab in breast cancer patients was inconclusive, possibly due to low expression of cMET in the trial patients [19]. Clinical support for the dual inhibition concept may be provided by the activity of cabozantinib, a tyrosine kinase inhibitor with selectivity, although not specificity, for inhibition of VEGFR2 and cMET kinases which has been approved for treatment of metastatic medullary thyroid cancer [20] and advanced renal cell carcinoma [21]. However, the relevance of cabozantinib to provide clear support for the dual VEGF/cMET inhibition concept is questionable because it inhibits several TKs in addition to VEGFR2 and MET [22]. Indeed, this broad activity probably accounts for the high incidence of adverse events that tends to limit the use of cabozantinib in combination with other agents [20].

In summary, the potential benefit of VEGF- and HGF-pathway inhibition has so far not been clearly realized in the clinic. Nevertheless, dual inhibition of these pathways appears to continue to offer promise for improvement over individual pathway inhibition. More potent inhibition of angiogenesis might be expected but potentially the most important benefit could come from prevention of the development of resistance to VEGF inhibition as well as to chemotherapy or other targeted therapies.

MP0250 is a DARPin^®^ molecule which specifically inhibits HGF and VEGF and can thus produce simultaneous inhibition of the two key growth factor pathways [23]. In the studies presented here we have demonstrated antitumor activities of MP0250 superior to those of individual VEGF and HGF-blocking DARPin^®^ molecules in a range of preclinical models as well as potentiation of the antitumor activity of chemotherapy and immunotherapy agents. MP0250 is the first target-specific bio-therapeutic agent acting on tumor and stroma simultaneously and its target specificity is expected to give it better tolerability, for instance, compared to the multi-targeted TKI cabozantinib.

## RESULTS

**Figure 1:**
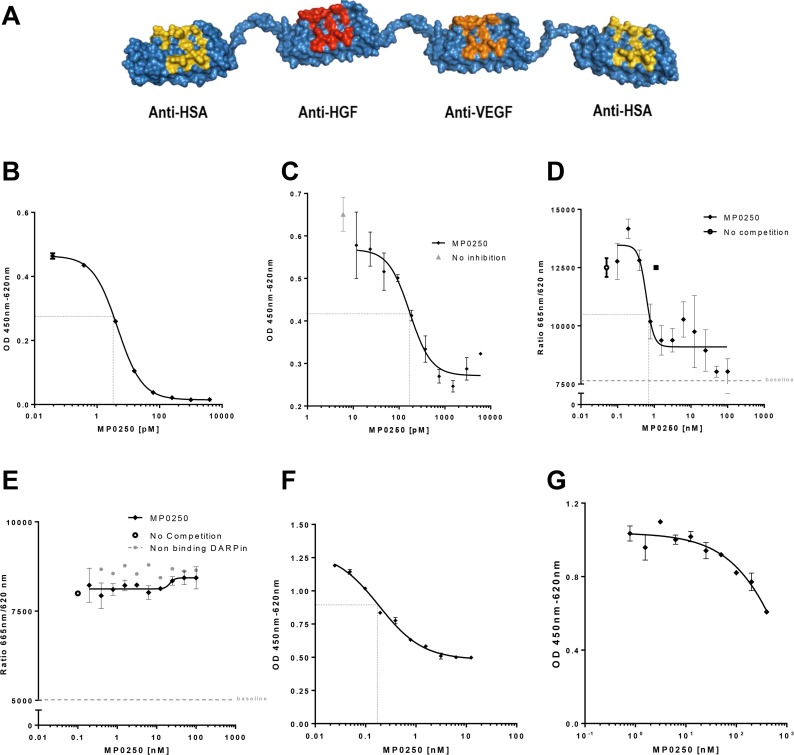
MP0250 and its Inhibition of VEGF- and HGF-induced cellular functions Model of MP0250, which is composed of two human serum albumin (HSA) DARPin^®^ molecules flanking a VEGF and a HGF binding DARPin^®^ molecule (**A**). The binding of MP0250 to VEGF was determined by quantification of free hVEGF in solution after addition of increasing concentrations of MP0250 (**B**). Inhibition of VEGF-induced HUVEC proliferation was analyzed in the absence (grey triangle) or presence of increasing concentrations of MP0250. Cell growth was quantified by OD measurement representing BrdU uptake. Error bars indicate the standard deviation of independent duplicates (**C**). Competition of binding of VEGF-A to sVEGFR2-Fc (**D**) and sVEGFR1-Fc (**E**) in the presence of increasing concentrations of MP0250 (filled diamond). As a control, a non-binding DARPin^®^ molecule was titrated (filled grey circle).The HTRF signal was detected. Error bars indicate the standard deviation of independent triplicates (1d, 1e). Inhibition of cMET-phosphorylation in A549 cells by MP0250. Inhibition of phosphorylation as measured by ELISA measurement (OD450-620) versus the concentration of the inhibitor (**F**). Inhibition of U87MG proliferation by MP0250; error bars indicate the standard deviation of independent duplicates (**G**). Dashed black lines in the figures indicate IC50s. Data shown in the figure represents one experiment out of independent experiments as outlined in the Materials and Methods section.

MP0250 is a dual-acting DARPin^®^ molecule containing VEGF-A and HGF binding domains (Figure 1A). Both domains are cross-reactive to the respective human and mouse growth factors and bind the growth factors of human and mouse with similar affinity in a sub-picomolar range [23].

### MP0250 inhibits VEGF-A-induced VEGFR2 signaling and endothelial cell proliferation

The VEGF neutralizing function of MP0250 was tested in a variety of functional *in vitro* assays. First, the potency of binding to recombinant human VEGF-A by MP0250 *in vitro* was determined with a sensitive quantitative sandwich ELISA. MP0250 showed a dissociation constant (K_D_) of 4.5 pM (Figure 1B). Next, neutralization of VEGF-A-induced proliferation of HUVECs was tested. To this end, proliferation of cells was induced with VEGF-A at a half-maximal effective concentration (EC50) of 3–5 ng/mL, equivalent to 71–120 pM human VEGF-A165 dimer. MP0250 neutralized the induction of proliferation of HUVECs with an IC50 in the range of 100–200 pM (Figure 1C). As induction of HUVEC proliferation by VEGF-A is mediated by VEGFR2 downstream signaling, a receptor competition experiment was performed to confirm that inhibition of endothelial cell proliferation by MP0250 is due to blocking of the VEGF-A / VEGFR2 interaction. MP0250 was shown to inhibit binding of VEGF-A to VEGFR2 with an IC50 of 0.6 nM (Figure 1D) but did not interfere with binding of VEGF-A to VEGFR1 (Figure 1E), most likely because different epitopes of VEGF interact with VEGFR2 and VEGFR1 [24].

### MP0250 inhibits HGF-induced cMET signaling and tumor cell proliferation

MP0250 was tested in HGF-dependent cellular response models to characterize the neutralization of HGF-mediated functions. First, inhibition of HGF-mediated cMET phosphorylation was tested in tumor cells *in vitro*. To this end, A549 tumor cells were stimulated with HGF (1 nM) in the presence of increasing amounts of MP0250 and phosphorylation of cMET was quantified with a phospho-tyrosine-specific cMET ELISA. MP0250 inhibited cMET phosphorylation with an apparent IC50 between 0.1 and 1 nM (Figure 1F). Next, inhibition of proliferation of the HGF-autocrine tumor cell line U87MG [25] was determined *in vitro* (Figure 1G). MP0250 inhibited proliferation of U87MG cells with an IC50 estimated at ~ 1nM from a non-sigmoidal inhibition curve (Figure 1G).

### MP0250 inhibits tumor growth in HGF- and VEGF-driven xenograft models

**Figure 2:**
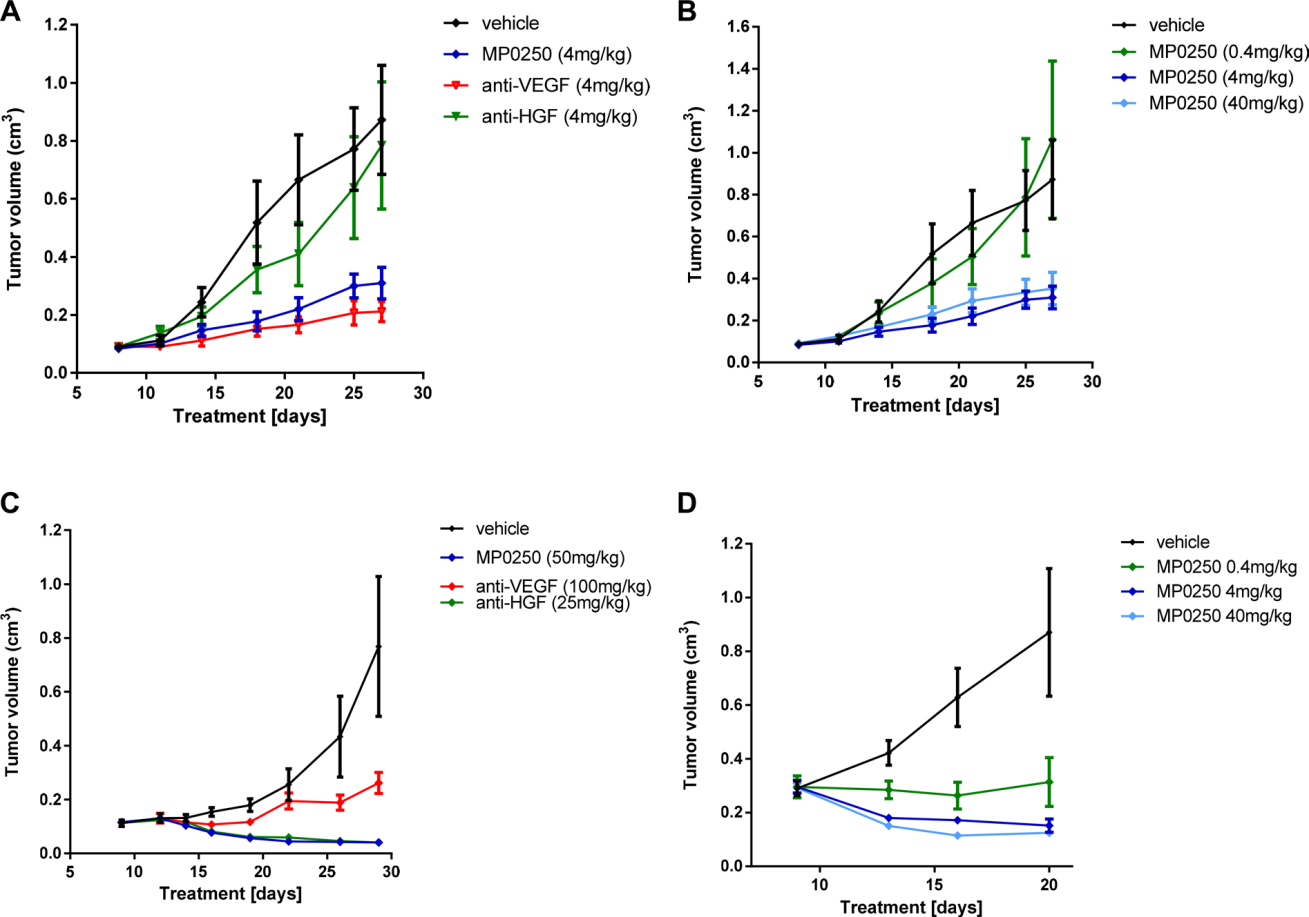
Tumor growth inhibition in U87MG and A673 xenograft models Tumor growth inhibition in the A673 rhabdomyosarcoma xenograft model (**A, B**) and the U87MG glioblastoma model (**C, D**). Figures 2A and 2C show the anti-tumor response to MP0250, the anti-HGF DARPin^®^ molecule and the anti-VEGF DARPin^®^ molecule. Figure 2B and 2D show a dose response of MP0250. Tumor growth is plotted as mean +/- SEM.

Mouse xenograft studies were performed to test whether MP0250 was capable of inhibiting the growth of human tumors. Thus, MP0250 was tested in the VEGF-A dependent A673 model and the HGF-dependent U87MG tumor model [25] [26]. In dose-response experiments, maximum antitumor activity was achieved at 4 mg/kg in both models (Figure 2B, 2D). In a further study in the A673 model, the antitumor activity of MP0250 (4 mg/kg) was compared to that of the same dose of DARPin^®^ molecules containing the individual inhibitor domains. MP0250 significantly inhibited tumor growth (35.5% T/C, *p* = 0.0139) to a similar extent to the VEGF-inhibiting DARPin^®^ molecule ACO279 (Figure 2A, Supplementary Table 1) while the HGF inhibitor ACO278 had no effect. In the U87MG model, MP0250 induced regression of U87MG tumors to a similar extent to the HGF inhibitor (both 5.3% T/C, *p* = 0.014). The VEGF inhibitor also had an anti-tumor effect in this model, although to a lesser extent (34.1% T/C, *p* = 0.075) (Figure 2C; Supplementary Table 1). These experiments show that MP0250 is capable of inhibiting both VEGF- and HGF-mediated functions *in vivo*.

Pharmacokinetic analyses showed that the mice had comparable exposure to the three molecules: serum half-lives of MP0250, ACO278 and ACO279 were 29 h, 30 h and 19 h respectively (data not shown).

### MP0250 inhibits tumor growth in syngeneic models

Monitoring HGF-mediated functions on tumor growth in xenograft models is limited by the fact that murine (stromal) HGF is not able to efficiently induce cMET signaling in human tumor cells [27]. The effect of HGF inhibition thus only reflects effects on functions of the HGF/cMET pathway in the tumor stroma if the implanted human tumor cells express human HGF which produces autocrine activation of cMET expressed by the cells. This is the case for U87MG tumors, as shown by the very potent inhibition of tumor growth by MP0250 (Figure 2C). This complication does not apply to VEGF as stroma-derived murine VEGF does activate human VEGF receptors [26].

**Figure 3:**
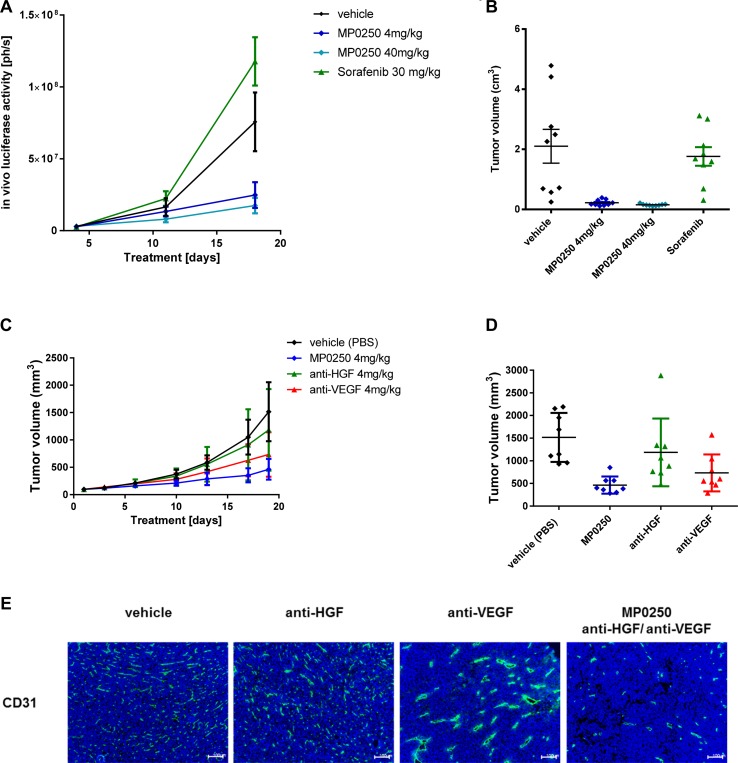
Tumor growth inhibition in syngeneic models and anti-angiogenic effect of MP0250 Tumor growth inhibition in the orthotopic renal cancer model (RENCA-LN model) (**A, B**) and the MC38 colorectal cancer model (**C, D**). Luciferase-transfected RENCA cells were orthotopically implanted into the left kidney of BalbB mice. Tumor growth was monitored by detection of luciferase activity *in vivo* during the study (Figure 3A) and determination of tumor volume at the end of the study (Figure 3B). MP0250 was compared to sorafenib at doses indicated in the figures. Figure 3C shows the time course of the anti-tumor response to MP0250 and the HGF inhibitor and the VEGF inhibitor. Figure 3D shows the tumor volumes at the end of the study. (**E**) shows the anti-angiogenic effect of the compounds in the MC38 effect demonstrated by immuno-histochemistry for CD31. Tumor growth is plotted as mean +/− SEM.

MP0250 binds both human and murine HGF and VEGF [23], therefore MP0250 was tested in two syngeneic mouse models, RENCA-LN and MC38. The efficacy of MP0250 was compared to the efficacy of the TKI sorafenib which inhibits VEGF receptor activation and thus VEGF-dependent functions in tumor growth. MP0250 showed a very strong anti-tumor effect and induced complete regression of tumors (10.6% T/C, *p* = 0.008) (Figure 3A, 3B; Supplementary Table 1). In contrast, sorafenib showed no anti-tumor effect in the model.

MP0250 also inhibited tumor growth in the second syngeneic mouse model, MC38 (31% T/C, *p* = 0.001; Supplementary Table 1). In comparison, the mono-inhibitory DARPin^®^ molecules neutralizing VEGF-A and HGF had T/Cs of 48% (*p* = 0.056) and 78% (*p* = 0.32) respectively. The increased efficacy of MP0250 over the individual inhibitors suggests an additive effect of VEGF and HGF blockade (difference MP0250 to VEGF-A DARPin^®^ molecule *p* = 0.028; MP0250 to HGF DARPin^®^ molecule *p* = 0.021) (Figure 3C,3D, Supplementary Table 1). This is not only reflected by inhibition of tumor growth but also by the strong anti-angiogenic effect of MP0250 (Figure 3E). Immunohistochemistry for blood vessels (CD31) showed that anti-HGF had no effect on micro-vessel morphology and density whereas anti-VEGF reduced the micro-vessel density to 80% compared to the vehicle group and promoted blood vessel normalization evident by vessels with a larger lumen and a stronger CD31 staining (Figure 3E). MP0250 showed the strongest anti-angiogenic effect; the number of blood vessels was markedly reduced to 40% of the vehicle group and blood vessels had a smaller lumen than in the anti-VEGF treated tumors, pointing to vessel regression rather than normalization.

### MP0250 inhibits tumor growth in patient-derived xenograft models

**Figure 4:**
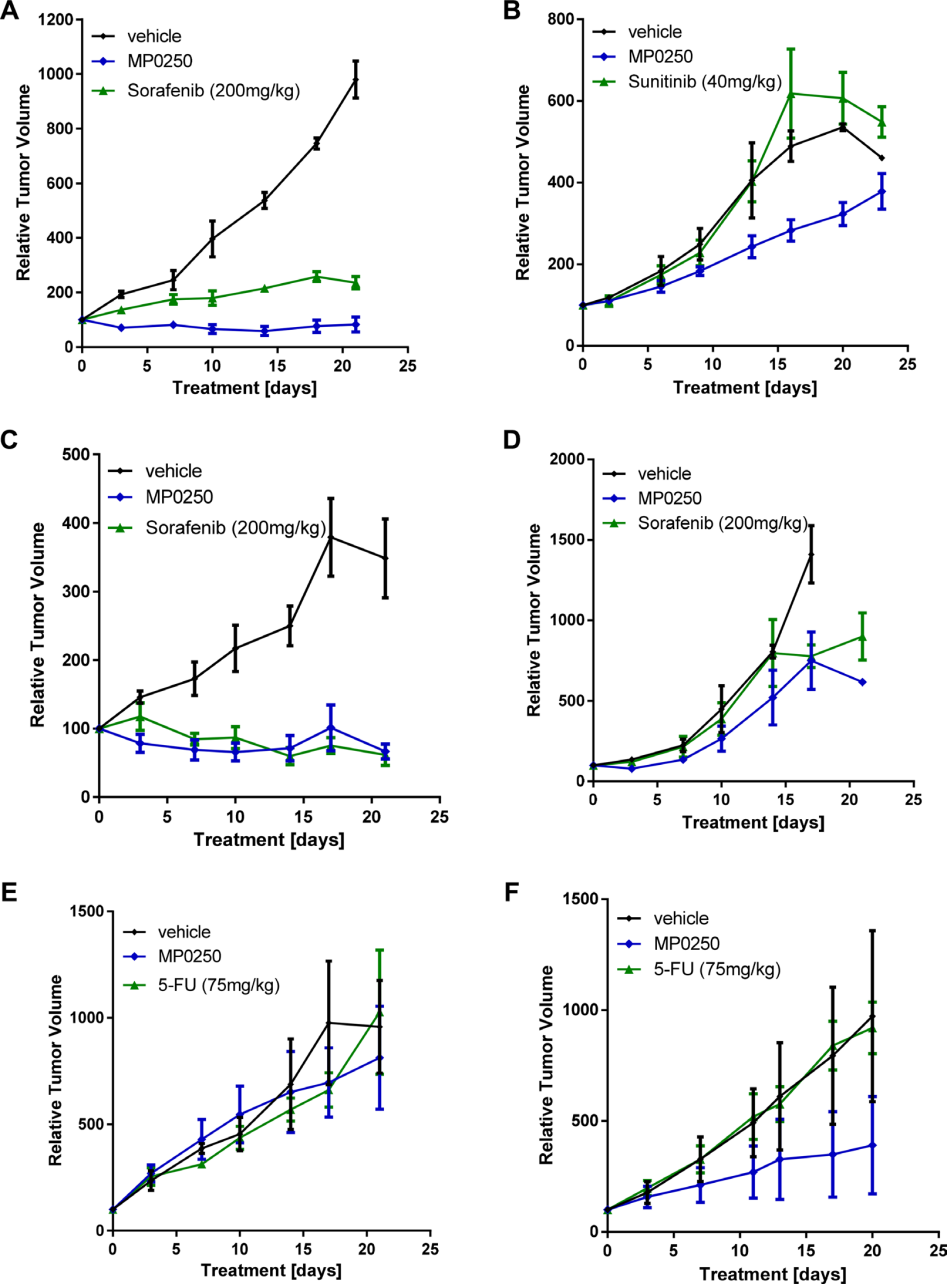
Tumor growth inhibition in patient-derived xenograft models Tumor fragments from two renal cell cancers, RXF2264 (**A**) and RXF616 (**B**), two liver cancers, LIXF658 (**C**) and LIXF575 (**D**), two lung cancers, LXFS650 (**E**) and LXFL1121 (**F**), were implanted into nu/nu mice. MP0250 was dosed at 4 mg/kg 3x weekly (i.v.); sorafenib at 200 mg/kg daily (p.o.), 5-FU at 75 mg/kg 1x weekly (i.p.), and sunitinib at 40 mg/kg daily (p.o).

**Figure 5:**
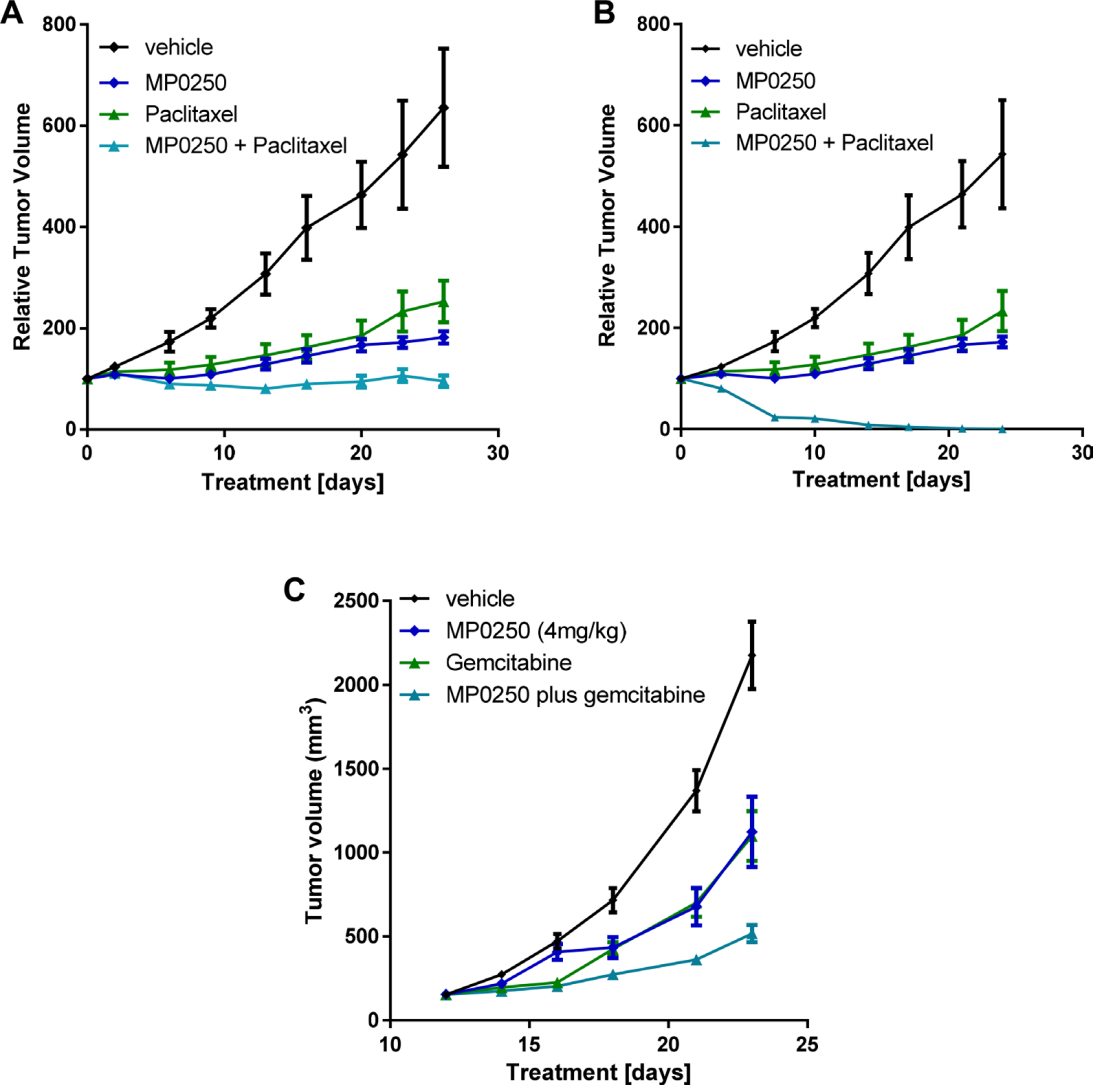
Tumor growth inhibition by MP0250 in combination with chemotherapy For combination therapy with paclitaxel tumor fragments from two gastric PDX tumors, GA3002 (**A**) and GA3027 (**B**), were implanted into nu/nu mice. For combination therapy with gemcitabine, KP4 tumor cells were implanted into nu/nu mice (**C**). MP0250 was dosed at 4 mg/kg 3x weekly (i.v.); paclitaxel at 15 mg/kg 1x weekly (i.v.). The combination of MP0250 and paclitaxel was dosed as 4mg/kg MP0250 3x weekly (i.v.) and 15 mg/kg paclitaxel 1x weekly (i.v.). The combination of MP0250 and gemcitabine was dosed as 4 mg/kg MP0250 3x weekly (i.v.) and 100 mg/kg gemcitabine 1x weekly (i.v.). Tumor growth inhibition of PDX models and of KP4 tumors is plotted as mean +/− SEM.

The efficacy of MP0250 in patient-derived xenograft models was tested and compared to standard-of-care drugs used in the clinic for the particular tumor types tested. Tumors were selected based on HGF expression levels (Supplementary Table 2); the majority of the models expressed HGF and were therefore assumed to be HGF autocrine. Parts of the study (renal, lung and liver models) were performed as a pilot study using three mice per group. However, marked anti-tumor effects with MP0250 were achieved (Figure 4; Supplementary Table 2). MP0250 showed anti-tumor activity in most models investigated (Figures 4 and 5). Its efficacy was superior to sorafenib and sunitinib respectively in both renal models RXF616 and RXF2264 (Figure 4A, 4B), and similar to sorafenib in both liver models LIXF658 and LIXF575 (Figure 4C, 4D). MP0250 showed similar anti-tumor activity to paclitaxel in both gastric cancer models (Figure 5A, 5B). MP0250 was more active than 5-FU in one lung cancer (NSCLC) model LXFL1121 (Figure 4F) but neither MP0250 nor 5-FU showed activity in the SCLC lung cancer model LXFS650 (Figure 4E). Tumor regression was induced by MP0250 in one liver (LIXF658) and one renal model (RXF2264) with optimal T/C values of 6.9% (*p* = 0.0024) and 8.9% (*p* = 0.0.0083). Significant tumor growth inhibition was recorded for both gastric cancer models (T/C values of 31.7%, *p* = 0.0085 (GXA3002) and 22.2 %, *p* = 0.0156 (GXA3027)) and moderate (not statistically significant) growth inhibition was shown in a second liver model (T/C values of 37%, *p* = 0.46), one lung model (T/C values 26%, *p* = 0.086) and in the second renal cancer model (T/C value of 56%, *p* = 0.156). Taken together, this shows that MP0250 has potent anti-tumor activity in a wide range of patient-derived xenograft (PDX) models. Moreover, HGF-autocrine models appear to be more susceptible to MP0250, confirming the results obtained in the U87MG (Figure 1) and RENCA (Figure 3A, 3B) models.

### MP0250 potentiates chemotherapy in xenograft models

The efficacy of MP0250 in combination with standard-of-care chemotherapy was tested in two gastric cancer PDX models and one pancreatic cancer xenograft model (KP4). In both gastric models, the anti-tumor activity of the MP0250-paclitaxel combination treatment was superior to that of the individual agents (GXA3002 T/C values: MP0250 31.7% (*p* = 0.0085); paclitaxel 46% (*p* = 0.02), MP0250/paclitaxel 16.7% (*p* = 0.0021); GXA3027 T/C values: MP0250 22.2% (*p* = 0.0156); paclitaxel 15.8% (*p* = 0.032), MP0250/paclitaxel 0% (*p* = 0.0006) (Figure 5A and 5B, Supplementary Table 1). In the KP4 model, the efficacy of the MP0250/gemcitabine combination was superior to that of the individual agents (T/C values: MP0250 51.7% (*p* = 0.0023), gemcitabine 50.5% (*p* = 0.0006), MP0250/gemcitabine 23% (*p* < 0.0001) (Figure 5C, Supplementary Table 1). A similar finding was made in a multiple myeloma model where MP0250 potentiates the efficacy of the proteasome inhibitor bortezomib [28].

### MP0250 potentiates anti-PD1 treatment in a syngeneic model

**Figure 6:**
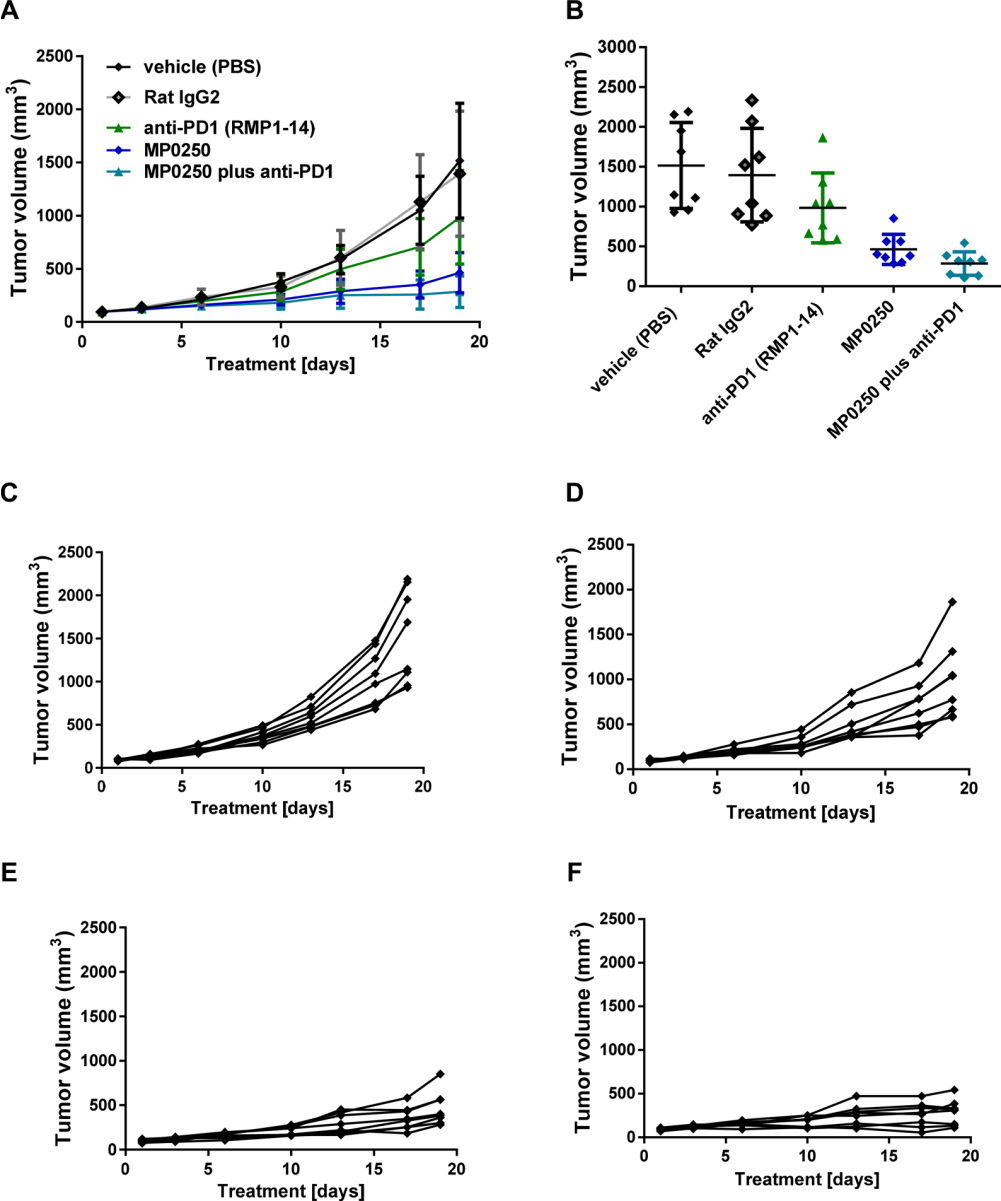
Tumor growth inhibition by MP0250 in combination with anti-PD1 MC38 tumor cells were implanted into C57/Bl6 mice. Mice were randomized into 5 groups of 8 mice. MP0250 was dosed at 4 mg/kg 3x weekly (i.v.); anti-PD1 antibody RMP1-14 was dosed at 5 mg/kg 3x weekly (i.p.). (**A**) shows the growth curve of the anti-tumor response, (**B**) shows the tumor volume at the end of the study. Tumor growth is plotted as mean +/- SEM. **(C-F)** shows the tumor growth curves of the individual animals; (**C**) shows the vehicle group, (**D**) the RMP1-14 treatment group, (**E**) the MP0250 treatment group and (**F**) the MP0250/RMP1-14 combination group.

In order to test whether MP0250 can increase the efficacy of anti-PD1 therapy in mice we performed a syngeneic mouse model (MC38) combining MP0250 with the anti-mouse-PD1 antibody RMP1-14. RMP1-14 produced moderate anti-tumor effects (T/C 65%, *p* = 0.048) whereas MP0250 showed a significant inhibition of tumor growth (T/C 31%, *p* = 0.0001) (Figure 3A, 3B, Figure 6A–6F). Interestingly, MP0250 potentiated the efficacy of the anti-PD1 antibody (T/C 19%, *p* < 0.0001) (Figure 6A, 6B). Three of 8 animals treated with a combination of anti-PD1 and MP0250 showed a reduction in tumor volume (%T/C < 10%) (Figure 6, Supplementary Table 1). This could not be achieved in the monotherapy arms. This study thus showed that MP0250 can potentiate immune therapy.

## DISCUSSION

It has long been recognized that VEGF pathway inhibitors give only relatively short-lived benefit in preclinical models and cancer patients [29] and studies have suggested that this may be overcome by concomitant inhibition of additional pathways [5, 30, 31]. Of these, the HGF/cMET pathway has seemed an attractive target as it has been shown to operate in many tumors, for example, preclinical studies in glioblastoma [25] and pancreatic neuroendocrine tumor models [32] have shown that MET inhibitors can overcome resistance to anti-VEGF therapy. However, a significant problem hindering clinical investigation of dual VEGF/HGF inhibition is that, to-date, no specific inhibitor of HGF or activation of cMET has demonstrated clinical benefit in pivotal trials [14] [15].

In an attempt to overcome the limitations of the currently available agents, we have created MP0250, a DARPin^®^ molecule that binds with high specificity to VEGF-A and HGF and neutralizes both activities with high potency [23]. The present study has demonstrated that the dual growth factor inhibition produced by MP0250 results in greater effects on tumor growth and angiogenesis than is achieved by inhibition of either of the growth factors individually in a wide range of tumors in syngeneic, cell-line-derived xenograft, patient-derived xenograft and orthotopic preclinical models (Figures 2, 3, 4). Importantly, MP0250 has also been shown to potentiate the activity of cytotoxic and immunomodulatory agents (Figures 5 and 6).

The high affinity binding of MP0250 to its target ligands was shown *in vitro,* using VEGF- and HGF-dependent cells, to produce potent inhibition of the separate functions of VEGF and HGF (Figure 1). This was extended into *in vivo* studies in which the growth of VEGF-dependent A673 tumors and HGF-dependent U87MG tumors was inhibited to similar extents by MP0250 and the individual VEGF and HGF inhibitory DARPin^®^ molecules respectively (Figure 2). Some insight into the relative contributions of VEGF and HGF to tumor growth was facilitated by the cross-reactivity of MP0250 as it inhibits human and murine HGF and VEGF with comparable potency. In the syngeneic MC38 model, treatment with either the anti-VEGF or the anti-HGF DARPin^®^ molecules inhibited tumor growth but MP0250 gave significantly greater inhibition (Figure 3), the implication being that both growth factors contribute to the growth of this tumor and its vasculature. In contrast, in the syngeneic RENCA model, MP0250 showed stronger anti-tumor activity than the VEGFR inhibiting kinase inhibitor sorafenib, implicating a more critical role for HGF than VEGF in the growth of these tumors. However, it is not known in either of these tumors whether the growth factors were of stromal or tumor origin. Interpretation of the relative roles of HGF and VEGF in xenografts is complicated by the fact that mouse stromal cell derived HGF does not efficiently activate human cMET and therefore makes no contribution to tumor growth. Instead, interpretation of HGF inhibition by MP0250 in xenografts is reliant on expression data to determine whether or not the tumors are likely to be HGF autocrine. In fact the HGF-autocrine U87MG xenograft (Figure 2C, 2D) and PDX models that were assumed to be HGF-autocrine were strongly inhibited by MP0250 (Figure 4 and Supplementary Table 2).

Of particular note from the histological examination of tumors was the markedly different effect of MP0250 on angiogenesis compared to the individual VEGF and HGF inhibitor DARPin^®^ molecules. The anti-HGF DARPin^®^ molecule had little discernible effect on tumor blood vessels but MP0250 gave a marked reduction in the number of vessels and the vessels present appeared to have no lumen. This was different from blood vessel normalization that has observed in anti-VEGF DARPin® molecule treated tumors and that has been described for purely VEGF blocking molecules such as bevacizumab [33, 34]. This difference could make a significant difference to tumor therapy as it is still under debate whether blood vessel normalization by anti-VEGF agents is beneficial for patients, e.g. by supporting delivery of standard-of-care drugs or is detrimental by supporting tumor growth [35].

MP0250 has a powerful anti-tumor effect as monotherapy and also potentiates the effect of paclitaxel in gastric cancer models (Figures 5A, 5B) and gemcitabine in a pancreatic cancer model (Figure 5C). These data clearly indicate the potential of combining MP0250 with chemotherapy, but also with other specifically targeted therapeutics, for example EGFR inhibitors (e.g. erlotinib) or B-RAF targeting molecules (e.g. vemurafenib). For both of these molecules it has been shown that over-expression or activation of the HGF/cMET pathway results in resistance and it has been shown in clinical trials that the HGF/cMET pathway is upregulated in patients who become resistant to anti-EGFR therapies [36] [37] [12]. Further, the enhancement of anti-PD1 efficacy by MP0250 (Figure 6) indicates the potential for combination therapy with the immune-therapeutic molecules that are currently revolutionizing cancer therapy [38]. The proposed mode-of-action of MP0250 in supporting immunotherapy is: (i) to support T-cell recruitment into the tumor by blocking VEGF and (ii) direct modulation of immune cell functions, including macrophages and dendritic cells [39] [40].

## MATERIALS AND METHODS

### Materials

Recombinant human VEGF-A, VEGFR1-FC and VEGFR2-FC were from Reliatech, human HGF and the polyclonal HGF neutralizing antibody were from RnDSystems. U87MG, A549, PC3 and DU145 cells were from ATCC or LGC. Primary human umbilical vein endothelial cells (HUVEC) were from Lonza. Cell culture media for culturing tumor cells were from LuBio and Lonza for HUVEC. Human VEGF Quantikine and P-cMET ELISAs were from RnDsystems. BrdU cell proliferation kit was from Roche.

MP0250 and DARPin^®^ molecules targeting HGF (ACO278), or VEGF-A (ACO279) were produced as described previously (Binz et al., 2017).

### Cell culture

HUVECs were grown in EGM-2/5% FBS containing supplement mix without VEGF-A. Cells were starved in EBM containing 5% FBS without supplement mix.

U87MG, A549 and DU154 cells were grown in DMEM containing 10% FBS.

### Cell proliferation assay

MP0250-mediated inhibition of VEGF-A-induced HUVEC proliferation was determined by titrating MP0250 in the HUVEC proliferation assay. Human VEGF-A was used at a concentration of 8 ng/mL. MP0250 was titrated from 200 ng/mL to 0.195 ng/mL. Cell proliferation was determined after 72 h by BrdU incorporation. The results were plotted using GraphPad Prism5 with a log (antagonist) versus response – variable slope (four parameters) equation. Four independent experiments have been performed.

Inhibition of U-87 MG cell proliferation was determined after 5 days of incubation with MP0250. Five independent experiments have been performed.

### cMET phosphorylation assays

200,000 A549 cells were seeded in complete medium which, was replaced by serum-free medium after 24 h. Cells were incubated for another 24h and stimulated by 1nM human HGF in the presence and absence of DARPin^®^ molecule. HGF and DARPin^®^ molecule were preincubated for 30 min at room temperature prior to addition to cells. Cells were stimulated for 10 minutes. Stimulation was terminated by removing the cell supernatant and addition of cell lysis buffer.

P-cMET levels in cell lysates were determined using the P-cMET-ELISA. Inhibition of cMET phosphorylation was calculated by setting the signal obtained in the non-stimulated control as 100% inhibition and the control without inhibitor as 0% inhibition. Five independent experiments have been performed.

### VEGF Quantikine ELISA

The assay was performed as described in the manufacturer`s protocol, except that the interference of VEGF binding to the capture antibody by MP0250 is analyzed. Non-neutralized VEGF (free-VEGF) is detected. The results were plotted using GraphPad Prism5 with a log (antagonist) versus response – variable slope (four parameters) equation.

### Receptor Competition Assay

DARPin and biotinylated VEGF-A_165_ (bioVEGF-A_165_) were pre-incubated for 1 h at room temperature. Human VEGFR1-Fc or VEGFR2-Fc were added and incubated for an additional hour. The formation of the VEGF-A-VEGFR-Fc complexes was monitored by HTRF using Streptavidin-Tb and PAb anti-hIgG-de. Two independent experiments have been performed.

### Animal experiments

Animal experiments were performed at contract research organizations according to standard procedures and local animal welfare rules: U87MG and A673 models at EPO Berlin, MC38 and KP4 at CrownBio, RENCA at Proqinase and PDX models at Oncotest/CRL. Briefly, for xenograft models, tumor cells (2 × 10^6^ cells / mouse) were implanted subcutaneously into the right flank of NMRI nu/nu mice for U87MG, A673 and KP4 and into C57/Bl6 mice for MC38. Mice were randomized into groups when tumors reached an average volume of 150 mm^3^. MP0250, ACO278 and ACO279 were dosed in the U87MG, A673 and MC38 models every three days for 6 times. The PD-1 antibody RMP1-14 was dosed at 5 mg/kg twice a week for three times in the MC38 model. MP0250 was dosed every three days four times in the KP4 model and gemicitabine at 100 mg/kg once weekly for two times in the KP4 model. For the orthotopic RENCA model, 0.4×10^5^ RENCA-LN labeled cells were implanted in the kidney subcapsule of BalbC mice. Growth of the tumors was monitored using *in vivo* bioluminescence imaging. Tumor-bearing animals were randomized according to imaging.

For PDX models, 4–5 mm diameter tumor fragments were implanted subcutaneously and treatment was started when tumor volume was 100–120 mm^3^. Eight xenografts were selected for testing based on their HGF expression levels: liver cancers LIXF575 and LIXF658, non-small cell lung cancer LXFL1121, small cell lung cancer LXFS650, renal cancers RXF616 (non-clear cell) and RXF2264 (clear cell) and gastric cancers GA3002 and GA3027. Groups were eight for the gastric cancers and three for the other models. MP0250 was dosed at 4 mg/kg 3x weekly (i.v.); sorafenib at 200 mg/kg daily (p.o.), 5-FU at 75 mg/kg 1x weekly (i.p.), sunitinib at 40 mg/kg daily (p.o.), paclitaxel at 15 mg/kg 1x weekly (i.v.). The combination of MP0250 and paclitaxel was dosed as 4 mg/kg MP0250 3x weekly (i.v.) and 15 mg/kg paclitaxel 1x weekly (i.v.). Mice were treated for 21 days. Tumor growth was monitored by caliper measurement. Relative volumes of individual tumors (individual RTVs) for Day x were calculated by dividing the volume on Day x (Tx) by the volume on Day 0 (T0) multiplied by 100%. Tumor inhibition for a particular day (T/C in %) was calculated from the ratio of the median RTV values of test versus control groups multiplied by 100%.

### Immunohistochemistry analyses

Immunohistochemistry was performed on tumors collected at the end of the MC38 study. Samples were sectioned and stained for CD31 using anti-mCD31- BD # 550274.

### Statistical analysis

Data were presented as mean ± SEM for animal studies. The Student *t* test was used to assess the significance of the difference between means.

## SUPPLEMENTARY MATERIALS FIGURES AND TABLES


