# Supplementary material for: MP0250, a VEGF and HGF neutralizing DARPin® molecule shows high anti-tumor efficacy in mouse xenograft and patient-derived tumor models
Source: Oncotarget. 2017 Oct 11;8(58):98371–83. doi: 10.18632/oncotarget.21738 (PMC5716736; doi:10.18632/oncotarget.21738)
Supplement: Supplementary file 1 [file oncotarget-08-98371-s001.pdf]

MP0250, a VEGF and HGF neutralizing DARPin® molecule shows high anti-tumour efficacy in mouse xenograft and patient-derived tumour models

SUPPLEMENTARY MATERIALS

Table 1: Anti-tumor response in xenograft and syngeneic mouse models

Model	Type and Site of implantation	% T/C (tumor volume) p-values versus vehicle	Comparision groups
A673	xenograft subcutaneous	MP0250: 35.5% ($p = 0.0139$)<br>Anti-HGF: 89.8% ($p = 0.144$)<br>Anti-VEGF: 24.2% ($p = 0.027$)	
U87MG	xenograft subcutaneous	MP0250: 5.3% ($p = 0.0144$)<br>Anti-HGF: 5.2% ($p = 0.075$)<br>Anti-VEGF: 34,1% ($p = 0.0143$)	
Renca	syngeneic Orthtopic (kidney)	MP0250 (4 mg/kg): 10.6% ($p = 0.0085$)<br>MP0250 (40 mg/kg): 7.2% ($p = 0.0081$)<br>Sorafenib: 83.95% ($p = 0.61$)	
KP4	xenograft subcutaneous	MP0250: 51.7% ($p = 0.0023$)<br>Gemcitabine: 50.5% ($p = 0.0006$)<br>MP0250 plus Gemcitabine: 23% ($p = < 0.0001$)	
MC38	syngeneic subcutaneous	MP0250: 30.6% ($p = 0.0001$)<br>Anti-HGF: 78.1 % ($p = 0.32$)<br>Anti-VEGF: 48.1 % ($p = 0.056$)<br>RMP1-14: 64.9 % ($p = 0.048$)<br>MP0250 plus RMP1-14: 18.8 % ($p = < 0.0001$)	MP0250 vs. Anti-HGF p= 0.021<br>MP0250 vs Anti-VEGF $p = 0.028$<br>MP0250 vs MP0250 plus RMP-1 $p = 0.053$ (tumor volume); $p = 0.049$ (tumor weight)

Table 2: Anti-tumor response in PDX models in correlation with HGF and VEGF expression

Model	Tumor entity	HGF-expression (Affimetrix)	VEGFA-expression (Affimetrix)	% T/C MP0250 (day)	% T/C SoC (day)	% T/C MP0250 plus SoC
LIXF 658	Liver<br>Adeno carcinoma	high	High	6.9% (45)	14.1% (31)	n.a.
LIXF 575	Liver<br>Hepatocellular carcinoma	low	High	37.4% (17)	49.1% (17)	n.a.
LXFL 1121	Lung<br>Large cell	high	High	26.2% (34)	87.8% (7)	n.a.
LXFS 650	Lung (Metastasis Lymph Node)<br>Small cell	moderate	High	88.7% (24)	78.2% (7)	n.a.
RXF 616	Kidney (Metastasis Lung)<br>Hypernephroma	high	High	56.4% (13)	79.1% (9)	n.a.
RXF 2264	Kidney	high	High	8.9% (25)	24.0% (25)	n.a.
GXA 3002	Stomach (Asian)	low	High	31.7% (26)	46.1% (20)	16.7% (26)
GXA 3027	Stomach (Asian)	low	High	22.2% (24)	15.8% (24)	0% (24)

Tumor inhibition for a particular day (T/C in %) was calculated from the ratio of the median RTV values of test versus control groups multiplied by 100%.The table summarizes the efficacy of the individual treatments (% T/C) and the expression of VEGF and HGF as determined by Affymetix mRNA expression profiling. Expression data were provided by Oncotest/CRL
